# Age-gender normal values of native and post-contrast myocardial T1 relaxation times (lambda) on 1.5T and 3T using MOLLI: a multicenter, single vendor cardiovascular magnetic resonance study

**DOI:** 10.1186/1532-429X-16-S1-P23

**Published:** 2014-01-16

**Authors:** Darius Dabir, Nicholas Child, Ashwin Kalra, Islam Z Mahmoud, Toby Rogers, Rolf Gebker, Ananth Kidambi, Sven Plein, Andrew Jabbour, David M Higgins, Bernhard Schnackenburg, Tobias Schaeffter, Eike Nagel, Valentina Puntmann

**Affiliations:** 1 King's College London, London, UK; 2 Philips Healthcare, Guilford, UK; 3 Leeds University, Leeds, UK; 4 German Heart Institute Berlin, Berlin, Germany; 5 St Vincent University, Sydney, New South Wales, Australia

## Background

Myocardial T1 mapping is emerging as a promising means to non-invasively discriminate between normal and diseased myocardium. We have shown that T1 measurements perfromed conservatively within the septal myocardium are reproducible and accurate with excellent discriminatory ability between normal and abnormal myocardium. Using this approach we aimed to determine age and gender related normal values at clinically used field strengths, 1.5 Tesla (T) and 3T, in a multi centre and single vendor study.

## Methods

We recruited normotensive subjects with no cardiovascular risk factors, low pretest probability of cardiovascular or systemic disease, taking no regular medication and subsequently, a normal CMR study (normal LV volumes, mass and no LGE), underwent native and post-contrast T1 imaging with modified look-locker inversion recovery (MOLLI; 3,3,5) either at 1.5T or 3T (Achieva, Philips Healthcare, Best, The Netherlands). Parameters for native and post-contrast MOLLI were identical (FOV 320 × 320; TR/TE/flip-angle: 3.3 ms/1.57 ms/50°, interpolated voxel size 0.9 × 0.9 × 8 mm, phase encoding steps n = 166, HR adapted trigger delay, with 11 (3-3-5) phase sampling arrangements. Adiabatic pre-pulse was used to achieve complete inversion. Septal ROIs were automatically propagated across all eleven images in the MOLLI sequence with a prior image- co-registration step for motion-correction (figure).

## Results

**Figure 1:**
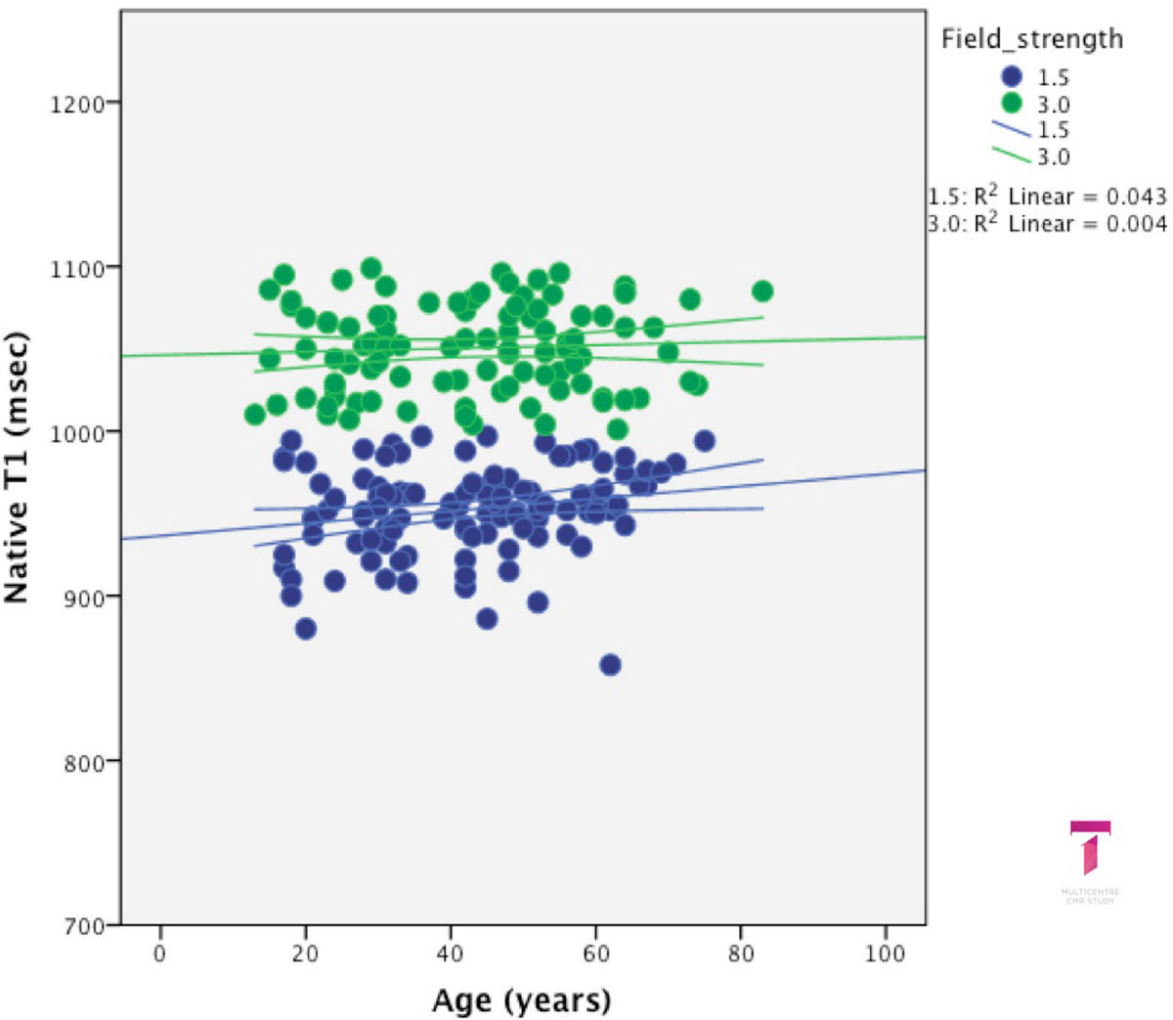


**Figure 2:**
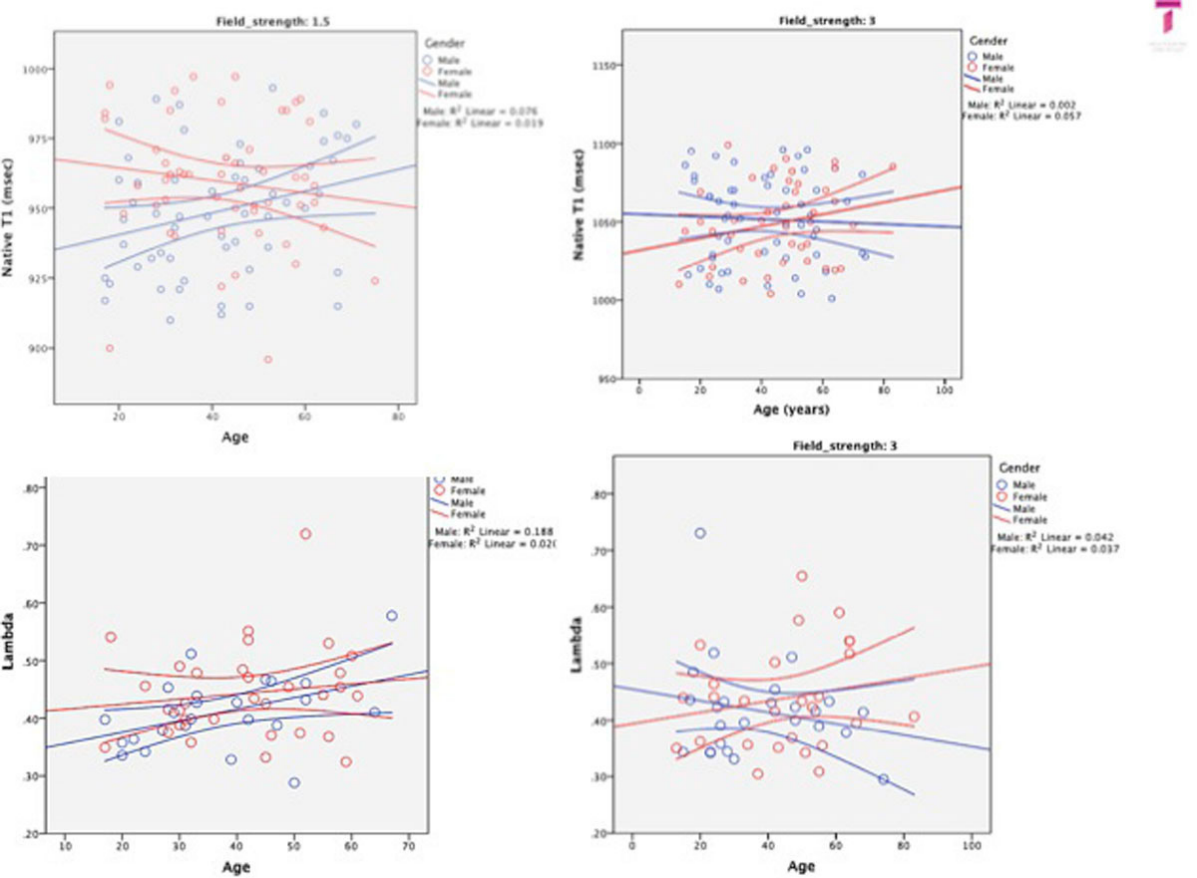


Two-hundred and thirty subjects were enrolled (age (years) median:43 (17-75), male: n = 118, 51%, 1.5T vs. 3T: n = 114 vs. 116. Mean (min-max; SD) T1 values (msec) per field strength were: native T1: 954 (896-997; 23) and 1052 (1001-1099; 27); post contrast T1: 400 (303-546; 59), lambda (%): 43 (29-72;9). There were no differences for gender or association with age for any of the T1 values or derivatives (Figure 1, 2).

## Conclusions

We report normal values for T1 values and derivatives, based on 3'3'5MOLLI sequence and using conservative septal sampling approach approach. We demonstrate no age or gender related differences at either field strengths.

## Funding

We would like to acknowledge Department of Health via the National Institute for Health Research (NIHR) comprehensive Biomedical Research Centre award to Guy's & St Thomas' NHS Foundation Trust in partnership with King's College London and King's College Hospital National Health Service Foundation Trust.

